# Genetic Diversity of Hepatitis E Virus Type 3 in Switzerland—From Stable to Table

**DOI:** 10.3390/ani11113177

**Published:** 2021-11-07

**Authors:** Isabelle Vonlanthen-Specker, Roger Stephan, Xaver Sidler, Dominik Moor, Cornel Fraefel, Claudia Bachofen

**Affiliations:** 1 Institute of Virology, Vetsuisse Faculty, University of Zurich, 8057 Zurich, Switzerland; isabelle.specker@outlook.com (I.V.-S.); cornel.fraefel@uzh.ch (C.F.); 2 Institute for Food Safety and Hygiene, Vetsuisse Faculty, University of Zurich, 8057 Zurich, Switzerland; stephanr@fsafety.uzh.ch; 3 Department of Farm Animals, Division of Swine Medicine, Vetsuisse Faculty, University of Zurich, 8057 Zurich, Switzerland; xaver.sidler@uzh.ch; 4 Risk Assessment Division, Federal Food Safety and Veterinary Office FSVO, 3003 Bern, Switzerland; dominik.moor@blv.admin.ch

**Keywords:** hepatitis E virus, one health, subtyping, Switzerland, pig, wild boar, food chain

## Abstract

**Simple Summary:**

The main hosts of hepatitis E virus (HEV) genotype 3 are porcine species. Transmission of the virus to humans, for example via undercooked meat, may cause acute or chronic hepatitis. To determine sources and routes of infection, comparing the viruses present in humans to the ones present in main hosts is a helpful tool. However, it requires knowledge of the genetic diversity of the circulating viruses. Therefore, we tested Swiss pigs and wild boars for HEV and determined the virus subtype and part of its genome. In addition, we determined the HEV subtype present in 11 positive meat products. One pig liver from the slaughterhouses (0.3%) and seven livers from a carcass collection (13%) as well as seven wild boar livers (5.8%) were found HEV positive. The same virus subtypes were found in Swiss pigs, wild boars, and meat products. Most of the viruses belonged to a Swiss-specific cluster within the subtype 3h. In addition, one pig liver and one wild boar liver were found positive for 3l and two meat products from Germany for 3c. Our data indicate that Switzerland has its “own” HEV viruses that circulate independent from the rest of Europe.

**Abstract:**

Hepatitis E caused by hepatitis E viruses of the genotype 3 (HEV-3) is a major health concern in industrialized countries and due to its zoonotic character requires a “One Health” approach to unravel routes and sources of transmission. Knowing the viral diversity present in reservoir hosts, i.e., pigs but also wild boars, is an important prerequisite for molecular epidemiology. The aim of this study was to gain primary information on the diversity of HEV-3 subtypes present along the food chain in Switzerland, as well as the diversity within these subtypes. To this end, samples of domestic pigs from slaughterhouses and carcass collection points, as well as from hunted wild boars, were tested for HEV RNA and antibodies. HEV positive meat products were provided by food testing labs. The HEV subtypes were determined using Sanger and next generation sequencing. The genetic analyses confirmed the predominance of a Swiss-specific cluster within subtype HEV-3h in pigs, meat products, and wild boars. This cluster, which may result from local virus evolution due to the isolated Swiss pig industry, supports fast differentiation of domestic and imported infections with HEV.

## 1. Introduction

The family of *Hepeviridae* includes two genera; the genus *Piscihepevirus*, which contains the single species *Piscihepevirus A,* also known as cutthroat virus of salmonids, and the genus *Orthohepevirus*, whose members infect mammals and birds and form the four genera *Orthohepevirus A, B, C* and *D* (https://talk.ictvonline.org/taxonomy, accessed on 5 October 2021). The most important species for human health is *Orthohepevirus A*, which is divided into 8 genotypes (HEV-1 to 8) [1,2,3,4]. The single-stranded positive-sense RNA genome is around 7.2 kb long and contains three open reading frames (ORF) of which the first and longest encodes the non-structural proteins, the second the capsid protein and the third a small protein that seems to be multifunctional and is, for example, associated with the release of the quasi-enveloped form of the virus [2,5].

In Europe, HEV-3 is considered the most important cause of locally acquired hepatitis [6,7]. In contrast to HEV-1 and 2, which are restricted to humans, HEV-3 is transmitted zoonotically. The main reservoir are porcine species with the domestic pig playing the most important role, but wild boars are also known to represent reservoir hosts [8,9]. Other animals such as deer and rabbits may also be sources of infection [10,11]. Several studies have indicated that pigs become infected early in life, once the maternal antibodies have sufficiently decreased. Three-month-old pigs seem to be the main virus shedders [12]. Virus is shed primarily via faeces for up to seven weeks, while viremia is usually more short-lived (1–3 weeks) but longer persistence in the liver is observed. Meat products containing liver are therefore considered a higher risk than muscular meat [13]. While naturally or experimentally infected pigs show no clinical signs and only histological changes in the liver [14,15,16], consumption of raw or undercooked meat, particularly liver, or direct contact with infected animals can lead to disease in humans [17,18]. The majority of infections have a subclinical or mild course. However, while no clear association to specific HEV-3 subtypes are observed, certain risk factors such as age over 50 and male gender, as well as immunosuppression, increase the risk for acute or, even more feared, chronic hepatitis, that may ultimately end in cirrhosis [19]. In addition, extra-hepatic manifestation such as neuralgic amyotrophy are frequently observed [20]. 

HEV has been reported in humans in several European countries since 2018, including Switzerland [21]. The seroprevalence observed in Swiss blood donors is with 20.4% comparable to other European countries, as is the percentage of antibody positive pigs (58.1%) and wild boars (12.5%) in studies from 2018 (humans) and 2014 (pigs and wild boars) [22,23]. Interestingly, in 2017 the full genome of HEV from a Swiss patient was determined and found to be of genotype 3, but with less than 88% nucleotide identity compared to published strains. It was therefore hypothesized to represent a potential “new” HEV-3 subtype [24]. In the same year we found closely related sequences in a human patient, the associated meat product and in a pig liver [25,26]. A recent publication has shown that this specific type of virus seems to be the most prevalent in Swiss patients [27]. However, the occurrence and diversity of HEV strains present in different potential sources of infection along the food chain in Switzerland is still unknown.

Molecular epidemiology has become an indispensable tool in determining routes and sources of infection in human and animal viral infections. Determination and comparison of viral variants, sero- or genotypes, has proven vital, not only in the current coronavirus pandemic but also in allowing us to link the new introduction of serotype 4 to emerging outbreaks of dengue virus in Indonesia, or recognize frozen berries as the source of a multistate outbreak of hepatitis A in Europe [28,29,30]. Furthermore, substantial sequence databases have helped tracing chains of (re-)infection of bovine viral diarrhea virus and supported the eradication of this epizootic cattle pathogen in Switzerland and Scotland [31,32]. Along this line, the European Centre for Disease Prevention and Control (ECDC) has initiated the HEVnet network in order to share molecular and epidemiological data on HEV globally and learn more about circulating HEV strains in Europe and the epidemiology of the virus [33]. However, the value and usefulness of such a platform, be it on the international or national level, depends on knowledge of the viral genetic diversity and epidemiological data. 

The aims of this study were therefore, (i) to gain further evidence for the potential presence of a Swiss specific HEV subtype and its prevalence in different hosts; (ii) to assess which other HEV subtypes are present; and (iii) to determine the extent of diversity within these subtypes. Therefore, we followed the food chain and sampled pigs of different ages, wild boars, and various meat products sold in Switzerland. We confirmed previous seroprevalence numbers for pigs and wild boars and found that the majority of viral genome sequences belonged to a genetic cluster of exclusively Swiss sequences within subtype 3h, formerly known as 3s(p). In contrast, common European subtypes such as 3c were only detected in imported meat products, indicating that HEV in reservoir species in Switzerland may circulate independently from the rest of Europe.

## 2. Materials and Methods

### 2.1. Samples

#### 2.1.1. Livers from Pigs at Slaughter

Liver samples from pigs at the timepoint of slaughter, which is normally at around six months of age in Switzerland, were collected between May and June 2018 in the three major pig slaughterhouses in Switzerland. Of the total yearly number of pigs slaughtered around 50% are processed in the slaughterhouses in Zurich, Courtepin and Basel (Personal communication R.S., March 2019) (a map indicating the location of the slaughterhouses is provided as supplementary Figure S1). In Zurich, 74 animals were sampled, in Basel 58 and in Courtepin 60. Additionally, 105 confiscated liver samples were collected by slaughterhouse staff members from Courtepin for this work. Confiscated livers are assigned as not being fit for human consumption, e.g., due to macroscopic lesions, such as parasite infections in the liver. The livers were individually packed in plastic bags, sealed, and stored at 4 °C for maximum one day until being transported to our Institute, where they were stored at −20 °C until further processing. Collection was performed over several weeks to sample multiple slaughtering batches.

#### 2.1.2. Livers, Feces, and Diaphragm from Pigs from Carcass Collection Points

Since pigs shed HEV mainly around three to four months of age, pigs younger than the slaughtering age are a more likely source of HEV. In addition, older animals may represent a reservoir for HEV. In Switzerland, animal carcasses below a weight of 200 kg must be disposed of in communal carcass collection points (CCP). In regions with a high density of pig farms, dead pigs of all ages, but most frequently young animals, can be found in these containments. Therefore, 54 animals of variable ages were sampled in two CCP in the canton Lucerne, more exactly in Hochdorf (*n* = 24) and Knutwil (*n* = 30), in March and August 2018 (Figure S1). The canton Lucerne is the main pig breeding and fattening area in Switzerland [23]. As sample material, a piece of the liver, the diaphragm and faeces from the colon were collected (in this order) on site from each animal individually. Different knives were used for each sample material and were disinfected for at least 15 min in 70% Ethanol and rinsed with hot water between different animals. The weight and age of the dead pigs was estimated. After transfer to our laboratory, the samples were labelled and stored at −20 °C before further processing. 

#### 2.1.3. Wild Boar Samples

From December 2017 to March 2019, a total of 75 liver samples were collected by hunting societies from the canton Schaffhausen (SH) in 14 different hunting grounds. Another 46 liver samples originated from the canton Ticino (TI) (Figure S1). These animals were shot in September 2018. The samples were individually packed in plastic bags, sealed, stored at 4 °C for max. 2 d and then transported to our laboratory where they were stored at −20 °C until usage.

In Switzerland, all wild boars meant for human consumption need to be tested for the zoonotic parasite *Trichinella spiralis* and therefore muscle tissue samples, normally from the diaphragm, are sent to one of the official testing laboratories by the hunters. What is left after testing is stored at −20 °C for a couple of weeks and then discarded. We used these archived samples, i.e., the meat juice available after defrosting the diaphragm samples, to test for HEV antibodies. The samples from the cantons Zurich (ZH) and Aargau (AG) were provided by the Institute of Parasitology of the University of Zurich, the samples from Schaffhausen (SH) by the Cantonal Veterinary Department Schaffhausen, and the samples from Basel-Landschaft (BL) and Solothurn (SO) by the Laboratory of Veterinary Diagnostics in Chur. In total 141 diaphragm samples from SH, 87 from AG, 64 from ZH, 92 from BL, and six from SO were received between November 2018 and June 2019. For some of the diaphragm samples (*n* = 55) no information regarding the origin of the samples was available.

#### 2.1.4. Meat Products

The diagnostic laboratory of the Federal Food Safety and Veterinary Office (FSVO) in Berne provided a total of 21 food samples that were tested positive for HEV between March 2016 and November 2018 [34]. From the Cantonal Laboratory of the canton Ticino, six HEV-positive mortadella di fegato sausages initially tested in 2016 and 2017 were supplied to our lab for genetic analysis of the virus. A list of all food samples included in this study is provided in Supplementary Table S1.

### 2.2. RNA Extraction

The QIAgen Viral RNA Mini Kit (Qiagen, Hombrechtikon, Switzerland) was used to extract the RNA from liver, faeces, diaphragm, and chunky meat products such as coarse sausages. For highly processed meat products such as liver patés, Trizol LS (ThermoFisher, Reinach, Switzerland) was used for RNA extraction. 

The viral RNA mini kit was performed according to the manufacturer’s instructions, using 140 μL input volume and 50 μL nuclease-free water for RNA elution. The following sample-type specific preparation methods were used. From the frozen liver and diaphragm tissue 30 mg were weighed in a 2 mL safe-lock Eppendorf tube. In the next step, 200 μL of nuclease-free water and a 5 mm steel bead (Qiagen, Switzerland) were added to the tube. Samples were then homogenized for 30 s at 25 Hz in the Tissue Lyzer II (Qiagen, Hombrechtikon, Switzerland). After a three-minute centrifugation step at 16,000× *g*, the supernatant was used in the QIAgen Viral RNA Mini Kit. For the RNA extraction from the faecal samples, 100 mg of faeces were weighed in a 2 mL Eppendorf tube and the 10-fold volume of phosphate-buffered saline (PBS) added to the tube. Samples were then homogenized for 30 s at 25 Hz in the Tissue Lyzer II (Qiagen, Hombrechtikon, Switzerland). After a 5 min centrifugation step at 16,000× *g* the sample was ready to be extracted. Salami-type sausages such as mortadelle were dissected manually to separate fat from meat/liver chunks. If these chunks were quite fresh, they were treated similar to raw tissue samples. If they were dry and hard (e.g., in salsiz sausages) 500 mg meat was soaked in 500 μL water in a 2 mL tube and pre-homogenized using the Tissue Lyzer without bead for 1 min at 25 Hz. In the next step, a 5 mm steel bead was added, and the soaked material homogenized for 1 min at 25 Hz. Of the resulting squish 100 mg was transferred to a new tube and, after addition of 200 μL of water and another steel bead, homogenized again in the Tissue Lyzer for 1 min at 25 Hz. After the subsequent 3-min centrifugation step at 16,000× *g*, the supernatant was used in the QIAgen Viral RNA Mini Kit. 

From the highly processed and fat-rich meat products such as liver patés, 200 mg were mixed with 700 μL of PBS in a 2 mL tube and a 5 mm steel bead was added. Homogenization was performed by running the Tissue Lyzer for 1 min at 25 Hz. After the samples were centrifuged for 3 min at 16,000× *g*, 250 μL of the supernatant were mixed with 750 μL Trizol LS and the RNA pelleted according to the manufacturer’s recommendation. The air-dried pellets were resuspended in 100 μL nuclease-free water (ThermoFisher, Reinach, Switzerland).

### 2.3. Real-Time RT-PCR

Real-time reverse-transcription PCR (rt RT-PCR) was performed on a Quant Studio 7 or Quant Studio 3 Real Time PCR System (Applied Biosystems, Waltham, MA, USA) following the protocol described by Garson et al. [35] which represents an adaptation of the PCR primers and probes originally described by Jothikumar et al. [36]. For the PCR reaction, the QuantiTect Probe RT-PCR Kit (Qiagen, Hombrechtikon, Switzerland) was used as recommended by the manufacturer. To control for successful RNA extraction porcine 12S rRNA was measured by real-time RT-PCR using the same cycling conditions and reagents as for HEV and using the forward and reverse primer (p12S_F 5′-CCACCTAGAGGAGCCTGTTCTATAA-3′; p12S_R 5′-GGCGGTATATAGGCTGAATTGG-3′) at 0.4 μM and the probe (p12S_P 5′-FAM-CGATAAACCCCGATAGACCTTACCAACCC-TAMRA-3′) at 0.2 μM. The RNA was added in a 1:100 dilution to the reaction (1 μL in 20 μL final reaction volume).

### 2.4. ORF2 Typing Nested RT-PCR and Sanger Sequencing

To determine the HEV genotype and subtype of positive samples a broad reactive nested typing RT-PCR was performed [37]. The first step in this protocol is to convert the viral RNA into cDNA. This was carried out using the RevertAid H-minus First Strand cDNA synthesis kit (Thermo Fisher Scientific, Reinach, Switzerland). The resulting cDNA was directly used in the first, outer PCR reaction, followed by the second, inner PCR reaction. In both steps the HotStarTaq DNA Polymerase (Qiagen, Hombrechtikon, Switzerland) was used following the recommendations of the manufacturer. Of the second PCR product, 5 μL were mixed with 1 μL loading dye and run on a 1.5% agarose-gel. If there was a clear, single band the rest of the PCR product (45 μL) was purified using the QIAquick PCR Purification Kit (Qiagen, Switzerland). The kit was used according to the manufacturer’s instructions, and DNA was eluted in 30 μL of the elution buffer included in the kit. If several bands were visible, the correct one was cut from the gel using a sterile scalpel blade, and DNA was extracted using the QIAquick Gel Extraction kit (Qiagen, Hombrechtikon, Switzerland). The DNA concentration of the sample was determined by the NanoDrop system (Thermo Fisher Scientific, Reinach, Switzerland). The forward and reverse sequencing primers were used for bi-directional sequencing (Microsynth GmbH, Balgach, Switzerland). After removing the primers, the sequence was 493 nucleotides long and part of the ORF2 of the HEV genome (position 5962 to 6454 of reference genome NC_001434). To determine the HEV geno- and subtype, the sequences were submitted to the online HEVnet typing tool (https://www.rivm.nl/mpf/typingtool/hev/, accessed on 10 August 2021) and phylogenetically analyzed. All sequences were submitted to the HEVnet sequence repository as well as to GenBank (accession numbers MZ923532-MZ923556). 

### 2.5. Next Generation Sequencing

Samples that were successfully subtyped by the ORF2 typing nested RT-PCR were subsequently subjected to next generation sequencing (NGS) to gain more information of the genome, ideally the full-genome sequence. Sample preparation and sequencing was performed following a method previously developed in our laboratory [38]. In summary, an enrichment for encapsidated viral nucleic acids was performed, followed by sequence independent single primer amplification and paired-end short read sequencing on an Ilumina NextSeq machine 500 with 2 × 150 bp read length for the majority of the samples. An Ilumina NovaSeq machine with 1 × 100 bp read length was used for one wild boar liver (WB74) and two meat products (BLV01185 and BLV01189). Quality control and screening to a database containing 61’620 complete viral genomes was performed as previously described [38]. Since the HEV genotype and subtype was already known from the ORF2 typing PCR based sequences, the NGS reads were subsequently aligned to HEV-3 subtype-specific databases containing all officially assigned full reference genomes of the respective HEV-3 subtypes [39] using the SeqMan NGen software from the DNAstar Lasergene Genomic suite (DNASTAR, Madison, WI, USA). The SeqManPro software was used to visualize the aligned reads and to generate and download the contigs. The contigs were blasted (https://blast.ncbi.nlm.nih.gov/Blast.cgi, accessed on 20 October 2021) to find the closest related publicly available HEV strain, and the reads were re-aligned and contigs generated against this reference alone using the SeqManPro software again. Finally, the complete and almost complete (>95%) contigs were screened for ORFs using the Clone Manager 9 Professional Edition software (Sci Ed Software LLC, Westminster, CO, USA). 

### 2.6. Phylogenetic Analysis

Phylogenetic analysis of the ORF2 sequences (493 nucleotide (nt) length) was performed using the MEGA X software [40]. After multiple sequence alignment by MUSCLE a Maximum Likelihood (ML) Tree with 1000 bootstraps, based on the Tamura-Nei model, was drawn. Besides the 26 own ORF2 sequences, the respective genome region of the recommended single representative of each HEV-3 subtype was included [39,41]. In addition, we added all HEV-3 reference genomes, as assigned by Nicot et al. [39], of the subtypes found in this study. However, while this was possible for 3h (*n* = 17) and 3l (*n* = 6) the total number of reference genomes appointed to 3c (*n* = 117) was too high for optimal visualization of the tree. Therefore, we selected three reference genomes most closely related to each of our two 3c sequences for the final tree (*n* = 6). 

For the four NGS derived complete and almost complete genome sequences the same single full-length representatives were included in the ML tree but only the 17 references for 3h were included as the NGS derived genomes all belong to subtype 3h. After alignment by MUSCLE, the full-length genomes were shortened to match the 5’ and 3’ ends of the partial genomes resulting in genome lengths varying between 6738 and 7138 nt. The shortened sequences were re-aligned and used for the ML tree as described above. For all sequences the GenBank accession numbers (MZ923532-MZ923556) are indicated in the phylogenetic trees. 

Following the method described by Nicot et al. [39], pairwise genetic distances were calculated in MEGA X after MUSCLE alignment, including 532 (near) full-genome references encompassing all available genotype-3 sequences and a set of 29 non-HEV-3 references [41]. The subtype demarcation cut-off of 0.093 was applied to confirm subtype assignment and to compare genetic distances of the new full-genome sequences and different clusters within subtype 3h. For visualization by boxplots the NCSS 10 statistical software (NCSS, LLC, East Kaysville, UT, USA) was used.

### 2.7. Antibody Detection

All samples originating from pigs and wild boars were tested for antibodies against HEV with the PrioCHECK HEV Ab porcine ELISA Kit (Thermo Fisher Scientific, Reinach, Switzerland). This indirect ELISA is suitable for porcine serum and meat juice samples. We used it for juices gained after defrosting diaphragm (‘diaphragm juice’) and liver samples (‘liver juice’). The ELISA was performed according to the manufacturer’s instructions. Optical densities were read in an ELISA reader (Sunrise Tecan, Tecan group Ltd., Männedorf, Switzerland) at 450 nm with the reference filter set at 620 nm and results interpreted as described in the manual.

To statistically compare antibody prevalence of wild boars from different cantons, the NCSS 10 statistical software was used (NCSS, LLC, East Kaysville, UT, USA). A contingency table provided evidence for the over-all difference. Subsequent pairwise comparisons were carried out using Chi-square statistics. Two-sided *p*-values ≤ 0.05 were considered significant. Canton Solothurn was excluded from the Chi-square statistics due to the small sample size.

## 3. Results

### 3.1. Prevalence of HEV RNA and Anti HEV-Antibodies in Domestic Pigs and Wild Boars

**Figure 1:**
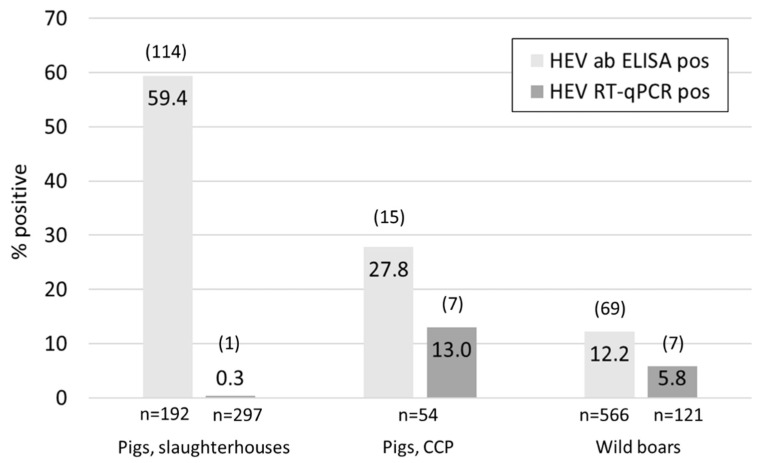
Overview of the percentages (numbers inside columns) and absolute numbers (in parentheses above columns) of HEV real-time RT-PCR (dark grey) and antibody positive (light grey) domestic pigs from slaughterhouses, carcass collection points (CCP), and wild boars from hunt.

Of the pig livers collected at the timepoint of slaughter, only one out of the 192 tested samples meant for human consumption was HEV positive (Figure 1). Additionally, 105 confiscated livers not fit for consumption were tested, but none of them contained HEV RNA. Overall, 59.4% of the tested samples were antibody positive, ranging from 46.7% in Courtepin (*n* = 60), 60.3% in Basel (58) to 68.9% in Zurich (*n* = 74). 

**Figure 2:**
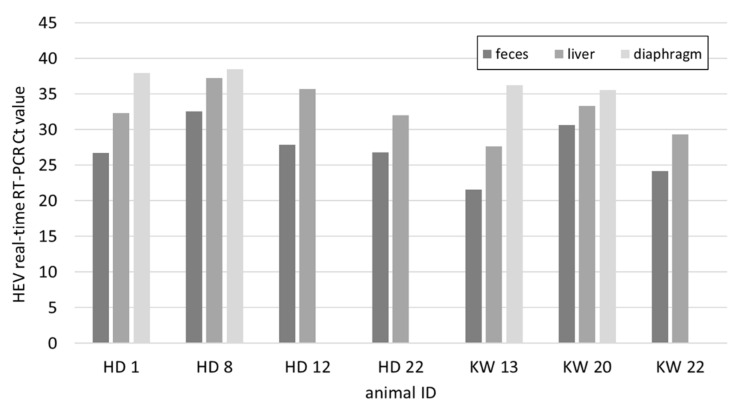
HEV real-time RT-PCR Ct values of faeces, liver, and diaphragm of 7 pigs from CCP that were determined RNA positive in the liver.

In the mainly younger pigs sampled at two carcass collection points (CCP) in Lucerne, seven out of 54 liver samples contained viral RNA and 27.7% of the animals were seropositive (Figure 1). The mean weight of all animals was 78.3 kg, while it was 42.1 kg for animals with an RNA positive liver and 76.3 kg for antibody positive animals (Table S2). From the seven positive animals the faecal and diaphragm samples were also tested for HEV. In all cases the faecal samples resulted in the lowest Ct values followed by the livers (Figure 2). The diaphragm samples tested positive in only four out of the seven pigs and had the highest Ct values. 

HEV RNA was detected in seven out of 121 tested liver samples from hunted wild boars in the canton Schaffhausen (SH) (*n* = 75) and Ticino (TI) (*n* = 46), resulting in an over-all RNA prevalence of 5.8% (Figure 1 and Table 1). However, all seven positive animals originated from SH; the RNA positivity was therefore 9.3% in this canton and 0% in Ticino. Regional differences were also observed regarding seroprevalence, as summarized in Table 1. In total, 566 liver and diaphragm samples were examined for antibodies and the overall percentage of positive animals was 12.7%. The highest seroprevalence (28%) was observed in the animals hunted in 14 hunting grounds in SH where we had a collaboration with the hunting societies and received fresh livers for PCR analysis. The second highest percentage (16.3%) was observed when testing the diaphragm juice from the *Trichinella* control in SH (44 hunting grounds), followed by the canton Zurich (ZH) (15.6%). The seroprevalence was considerably lower in animals shot in TI (6.5%), Aargau (AG) (4.6%) and Basel-Landschaft (BL) (7.6%). The differences were statistically significant between SH (total) and AG (*p* = 0.0004), SH and BL (*p* = 0.0068), SH and TI (*p* = 0.0323), and ZH and AG (0.0253). Information regarding the age of the animals was available for 68 of the 75 liver samples from Schaffhausen. The majority of the analyzed samples were from young boars (<1 year, *n* = 32), followed by juveniles (*n* = 19) and adults (>2 years, *n* = 17). Two of the seven RNA positive animals belonged to the group of young boars, three were juvenile and in two cases the age was unknown (Table S3). Of the 21 antibody positive animals, six were adults, nine juveniles and five young boars. Hence, the group of juvenile wild boars constituted the largest number not only of virus- but also of antibody positive animals. Interestingly, five of the seven RNA positive animals were also antibody positive: three juveniles, one young boar and one of unknown age (Table S3). 

### 3.2. HEV Subtyping by Sanger Sequencing

The subtyping PCR was successful for 15 out of 16 HEV positive samples from pigs and wild boars and for 11 out of 27 different meat products (Table 2, Table S1). According to the HEVnet online typing tool, all subtyped samples originating from Switzerland belonged either to the formerly proposed subtype 3s(p) or, in two cases, to the formerly proposed subtype 3o(p). According to the recently published demarcation cut-off for HEV-3 subtypes, 3s(p) and 3o(p) full-genome sequences are assigned to the established subtypes 3h and 3l, respectively [39]. The sequences of two meat products produced in Germany were clearly assigned to the subtype 3c. The sequences from liver and faeces of pig KW13 were identical, therefore only one was submitted to GenBank and used for phylogenetic analysis.

### 3.3. NGS and Phylogenetic Analyses

To gain more information on the genome of the viruses and confirm the subtyping result gained by the partial ORF2 sequence, NGS was performed on all samples where ORF2 sequencing was successful and sufficient sample material was available. The total read numbers after QC ranged between 3 and 10 Mio reads per sample, while the number of reads aligning to a HEV reference genome was highly variable as was the genome coverage (Table 2). The highest read numbers and best coverage was observed in the six wild boars with an average of 75.2% genome coverage, one complete sequence and two sequences with >95% coverage. No HEV reads were detected in the sample from the single positive pig from the slaughterhouse. In contrast, with exception of KW20, all samples from pigs from the CCP contained HEV reads. The average genome coverage was 64.9%, and one sample was nearly fully covered (KW13, 98%). In contrast, the meat products performed rather poorly in NGS. Of the seven samples included, four failed completely and the other two resulted in very low read counts and poor coverage of 1.4% and 23.4%. No clear correlation between Ct value and NGS performance was visible (Table 2). An overview of the coverage pattern is provided in supplementary Figure S2.

For two of the four sequences with >98% coverage, WB33 and WB40, all three ORF described for HEV were fully covered and coded for the expected number of amino acids. In case of WB36, 92 nt were missing at the 3’end, which leads to a truncated ORF2 product of only 657 instead of 660 amino acids. In contrast, the sequence of sample KW13 lacked 127 nt at the 5’end, resulting in an ORF1 product of only 1670 amino acids instead of 1703. The sequences of the three wild boars were nearly identical, with only a dozen of mismatches between them over the whole genome.

**Figure 3:**
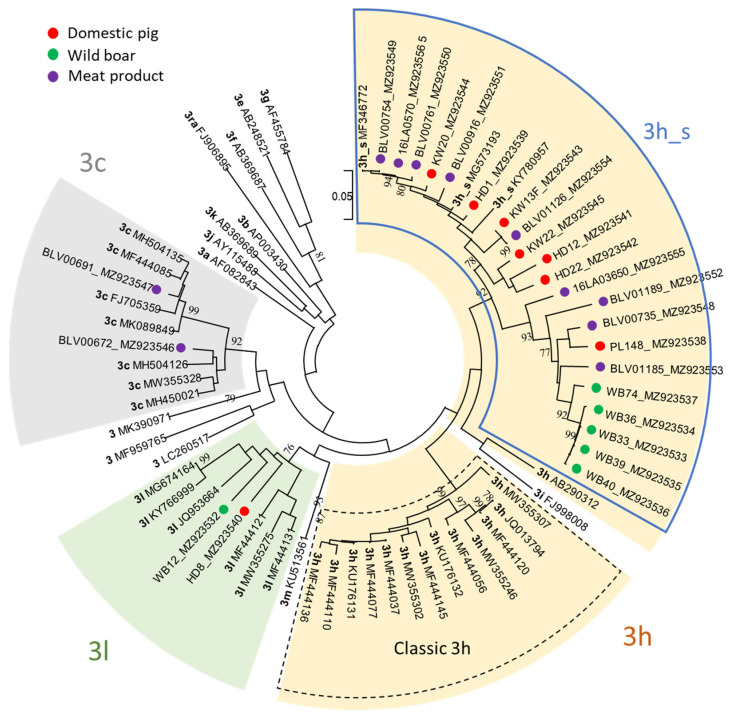
Phylogenetic tree of a 493 nt long fragment of the ORF2 including the 26 sequences resulting from this study (colored dots; accession numbers provided after sequence name) and 42 previously assigned HEV-3 reference genomes [39,41]. The evolutionary history was inferred by using the Maximum Likelihood method and Tamura-Nei model [42]. The tree with the highest log likelihood from 1000 bootstrap replicates is shown. The tree is drawn to scale, with branch lengths measured in the number of substitutions per site. Bootstrap values above 75% are shown. For the subtypes 3h and 3l all previously assigned genomes, for 3c a selection of six assigned genomes and for all other subtypes the single representative reference genomes as previously suggested [41] were included.

The ML tree based on the 493 nt long ORF2 fragment confirmed the subtype allocation by the HEVnet typing tool. The majority of sequences from pigs, wild boars and meat products (22 out of 26) grouped within subtype 3h and formed a well-supported monophyletic cluster, henceforth named 3h_s, clearly distinct from the classic 3h sequences (Figure 3). Only two sequences within 3h_s were found identical: KW13F and BLV01126, from a pig and a salsiz sausage, respectively, while all other 3h_s sequences differed for at least one nt. The tree showed also that the cluster 3h_s may be separated into two branches. Both contain sequences of domestic pigs and meat products, but only one includes official 3h reference genomes, while the other contains most of the wild boar sequences, which clustered closely together. Two sequences, one wild boar and one pig, were confirmed to belong to subtype 3l, as proposed by the online typing tool. The assignment of sequences from two meat products from Germany to subtype 3c was also confirmed, and they were shown not to be closely related.

**Figure 4:**
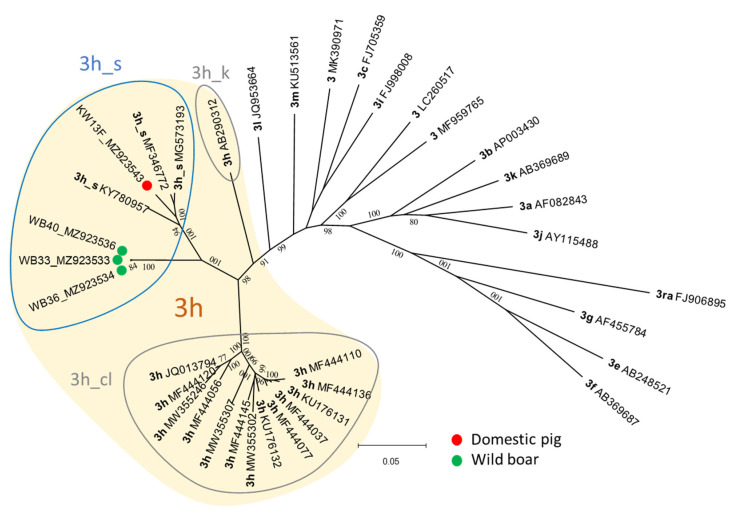
ML tree of the four NGS derived sequences (colored dots) and 32 HEV-3 reference genomes. For the subtype 3h all official references, for all other subtypes the single recommended representatives [39,41] were included. Where necessary, genomes were shortened to identical starting and ending nucleotides (6738–7138 nt length). The evolutionary history was inferred by using the Maximum Likelihood method and Tamura-Nei model [Tamura K. and Nei M. (1993)]. The tree with the highest log likelihood from 1000 bootstrap replicates is shown. The tree is drawn to scale, with branch lengths measured in the number of substitutions per site. Bootstrap values above 80% are shown. The distinct clusters within 3h, namely the classic 3h genomes, the formerly proposed subtypes 3s(p) and 3k(p) are encircled and named 3h_cl, 3h_s and 3h_k, respectively.

Another ML tree was calculated including the four complete and almost complete sequences (>98% coverage) originating from three wild boars and one pig from CCP. While the three sequences from the wild boars group very closely together, the sequence from the pig is a little further away, but all sequences clearly belong to the cluster of Swiss sequences within 3h, 3h_s (Figure 4). As already seen in the phylogenetic tree of the partial ORF2 sequences, the 3_s sequences form an own cluster containing two branches and are distinct from classic 3h sequences (3h_cl) and the former 3k(p) sequence (3h_k).

**Figure 5:**
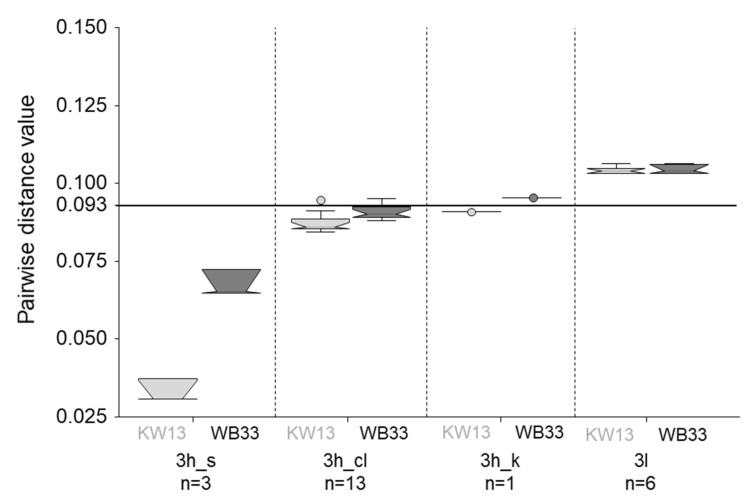
Boxplot visualization of the pairwise distance values of comparing KW13 (light grey) and WB33 (dark grey) full genomes to all full-length reference sequences of subtypes 3h and 3l. Separate distances were calculated for the three clusters within 3h that relate to the classic 3h genomes (3h_cl) and the formerly proposed subtypes 3s(p) (3h_s) and 3k(p) (3h_k) (Figure 4). The number of available references for each subtype/cluster are indicated below the names. The subtype demarcation cut-off of 0.093 is indicated by a horizontal line. The whiskers extend to 1.5x interquartile range (IQR) from the box edge. Outliers are marked by circles. The notches are constructed using the formula: Median +/− 1.57 × (IQR)/√n.

To quantify the genetic relatedness and confirm subtype assignment, pairwise-distances were calculated including 532 reference sequences from all subtypes of HEV-3, single representatives of genotypes one to four, and our four (near) full genomes. Only members of subtype 3h showed genetic distance values below the cut-off of 0.093 when compared to the new sequences, confirming assignment of all four genomes to this subtype (Figure 5, Table S4). Interestingly, KW13 was shown to be more closely related to the already published 3h_s reference genomes and to the classic 3h sequences than the wild boar representative, WB33 (Figure 5). The genetic distance values of WB33 are only just below the cut-off when compared to 3h_cl and above compared to 3h_k. The pairwise distance to 3l, the most closely related subtype after 3h, is nearly identical for WB33 and KW13 and clearly above the demarcation cut-off. 

## 4. Discussion

### 4.1. Prevalence Data

#### 4.1.1. Domestic Pigs

When screening 192 livers from the three largest pig slaughterhouses in Switzerland, only a single sample was virus RNA positive, resulting in a prevalence of 0.3% (Figure 1). This is somewhat lower but comparable in range to previous findings in Switzerland where 1.3% (two out of 160 livers) were found positive [13]. Individual RNA prevalence values may vary substantially between different studies and countries, with a range of 1–89% [12]. However, samples from pigs at slaughter tend to be relatively low, e.g., 4% in France and 3% in UK [43,44]. In contrast to the RNA prevalence, HEV-specific antibodies were found in 59.4% of the Swiss slaughterhouse samples. This finding confirms a previous observation of 58.1% seroprevalence in Swiss pigs at slaughtering age sampled in 2006 and 2011 [23] and underpins the finding that most pigs will become infected relatively early in life and have already developed an immune response and cleared the virus at the slaughtering timepoint. Early slaughtering timepoint and late infection, e.g., due to prolonged protection by high levels of maternal antibodies, were shown to be risk factors for pigs to be HEV positive at slaughter [45,46]. Seroprevalence of slaughtering pigs in Europe ranges between 30–98% [12]. The prevalence in Swiss pigs is therefore somewhere in the mid-range and seems to be rather stable over time. 

A much higher percentage, 13%, of the 54 tested animals from CCP was found virus positive in the liver. The weight of four of the seven positive animals was between 20 and 25 kg; the remaining three animals were estimated to be 50, 60 and even 100 kg (Table S2). These results confirm the findings of others, that the peak of viremia in pigs is around three to four months of age, but older, chronically infected animals, may serve as virus reservoirs [46]. In all seven cases, the diaphragm was found least positive, with Ct values of over 35 in four cases and a negative result in the three others. Similar findings were previously made in Spain [47] and are in support of the risk assessment of meat products that classify products containing pig liver as higher risk compared to porcine muscular meat [13]. 

#### 4.1.2. Wild Boars

In addition to domestic pigs, wild boars are known to be an important source of infection. Overall, we found 5.8% and 12.2% of RNA- and antibody positive animals, respectively. A nearly identical value for the antibody prevalence (12.5%) was found in samples collected by hunters of 10 different cantons in 2008, but no study on virus prevalence has been carried out yet and no HEV sequencing data was available of Swiss wild boars [23]. However, our data may not be representative for all of Switzerland. While we received diaphragm juice samples from most of the cantons with high wild boar density [48], this sample material is not ideal for detecting circulating viruses, as seen in the CCP samples. In addition, the low number of antibody positive animals in many cantons indicated that the likelihood to find HEV RNA positive samples among the 445 diaphragm juices was small. We therefore refrained from testing the diaphragm juice for viral RNA. However, we received 121 livers for RNA detection from two cantons, SH and TI, which are in the very north and south of Switzerland, respectively, and have both a high wild boar density. Interestingly, not a single liver of the 46 samples from TI was found HEV positive, in contrast to seven out of 75 in SH (Table 1). Significant differences were also observed in the percentage of antibody positive animals between different cantons, with higher numbers of around 15% in the east (SH and ZH) compared to more westerly cantons where it was around 4%. However, more samples also from the French speaking areas and more representative numbers of samples would be necessary to confirm this trend. In Germany, spill-over of HEV from wild boars to deer species was observed [49]. While we did not specifically investigate deer samples, none of the 14 incidentally provided roe deer livers from hunting grounds where HEV positive wild boars were identified were RNA or antibody positive (data not shown). Our data indicate that HEV may circulate in the wild boar population primarily within relatively small-scale geographic patterns and virus prevalence may vary from 0% to 26% (Table 1, Table S3, Figure S3). It was recently shown that HEV seroprevalence in wild boar populations are influenced not only by population density but also by environmental factors such as rainfall and proximity to marshland and may fluctuate considerably over time [50]. It is therefore impossible to draw conclusions on the whole wild boar population from geographically and temporally limited HEV data.

### 4.2. Sequencing Data

#### 4.2.1. Partial ORF2 Sequences

To determine the circulating HEV genotype and subtypes we used a 493 nt long partial ORF2 PCR product that has been described before [37]. We chose this method since it is widely used by national HEV reference and research laboratories, as shown by an interlaboratory HEV typing comparison test and, hence, facilitates international comparison of the sequences [51]. However, examples of recombination-events within the HEV genome have shown that relying on the partial ORF2 sequence alone may not always provide correct classification [52,53,54]. Therefore, to assign novel sequences reliably to the 11 official subtypes, ideally the full genomes should be used [39]. However, the full-genome Swiss sequences analysed to date have not provided evidence for recombination, and the partial ORF2 sequences resulted always in the same tree topography as the full-length sequences [25,26].

While nearly all positive samples from pigs and wild boars were successfully sequenced, only 40% of the meat products provided a sequence. It has been reported before that sequencing of meat products, particularly highly processed ones, is challenging, probably due to inhibitors and degradation of RNA [34,55]. While our methodology has previously been successfully used for full-genome sequencing of HEV from a mortadella pork sausage [25], further optimization of the RNA extraction method may be necessary for different product types. Importantly, the resulting RNA should be suitable not only for real-time RT-qPCR but specifically for sequencing, which, as our data show, not always coincides. 

Overall, 84.6% of the 26 sequences from this study were assigned to subtype 3s (p) (now part of 3h) by the HEVnet online typing tool and were confirmed by a ML tree. The tree clearly shows that the Swiss sequences form a distinct cluster, representing the former 3s(p) subtype, here named 3h_s. Until now, HEV-3h_s has exclusively been reported in Switzerland, not only in pigs, wild boars, and meat products, but also in humans [27]. This finding is quite remarkable as, to our knowledge, there are no other country-specific subclusters of HEV-3 reported that are predominant “from stable to table”. As stated previously, the unique situation in Switzerland is most likely attributed to a high degree of self-sufficiency regarding Swiss pork consumption and the fact that Switzerland is not part of the European Economic Area [27]. In England and Wales, for example, the sequences found in humans are more closely related to porcine sequences from mainland Europe rather than the UK; most likely due to a high degree of imported pork [56]. Furthermore, due to the high animal health status of pigs in Switzerland, the import of live animals is strictly limited. Additionally, Swiss pigs are only moved within Swiss borders, while pigs born in the European Union may be transported across borders, e.g., for fattening and/or slaughtering, which may contribute to the exchange of HEV subtypes. 

In one wild boar and one pig HEV-3l (former 3o(p)) was detected. This subtype seems to be relatively rare, only six annotated full-genome references are publicly available. Four of these originate from France (3× human, 1× porcine), the remaining two from Italy (2× porcine) [39,57]. Due to the limited number of 3l sequences, we cannot conclude on any geographical pattern within Switzerland. However, compared to 3h, in Swiss sequences form our own cluster, this is not the case for 3l, which speaks against geographical clustering. Subtype 3l sequences have also occasionally been detected in human patients in Switzerland and, hence, are probably not rare in Swiss pigs [27]. Interestingly, the wild boar positive for HEV-3l was shot in a hunting ground that directly borders Germany (Figure S3). It would be interesting to know the HEV genotypes present in the wild boar population in Southern Germany and if there are 3l or even 3h_s subtypes present.

We have not discovered any of the HEV-3 subtypes in Swiss pigs that are predominant in other European countries (i.e., 3c, 3e, 3f). However, we have analysed only a limited number of sequences. A broader screening of Swiss pig herds and wild boar, e.g., using faecal samples, would be necessary to also detect rarer HEV-3 subtypes. 

Overall, sequences of pigs and meat products are evenly distributed over two branches within 3h_s, while most wild boar sequences seem to form their own small cluster within one of the branches. However, this may be attributed to the fact that all wild boars originated from the same geographical area and most of them even from the same hunting ground (Table S3). Still, we can assume that the same HEV subtypes circulate in pigs and wild boars. Interestingly, a different situation was observed for atypical porcine pestivirus, where Swiss-specific sequences circulate only in domestic pigs [58], while wild boars harbour other, more “European” sequences (personal communication, Matthias Schweizer, Institute of Virology and Immunology, Bern, October 2021).

Direct comparison of our sequences to previously published human sequences is limited due to the different sequencing approaches used [27]. However, 58.9% of the HEV-3 sequences of human origin (*n* = 95) belonged to subtypes also detected in Swiss pigs (54.7% to 3s(p) and 4.2% to 3o(p)). In addition, HEV-3f (7.4%) and 3a (2.1%) were found in humans but so far not in Swiss pigs or wild boars. Interestingly, also 3ra, originating from rabbits (3.2%), was found in human patients. Another 28.4% of sequences could not be assigned due to the relatively short amplicon used for sequencing [27].

#### 4.2.2. NGS Derived Sequences

In addition to the partial ORF2 sequences, we subjected the samples to NGS in order to gain longer sequences, which would be helpful for molecular tracing. As expected, this has worked better for fresh sample material such as liver samples than for the meat products. Interestingly, the Ct value was not a reliable indicator of sequencing success. Nevertheless, in 30% of the samples, the coverage was over 80%, which allows more reliable characterization of the genome. Unfortunately, NGS of neither of the two samples containing 3l sequences resulted in high coverage. However, four 3h_s sequences were covered between 95.6% and 100% and could be compared to other (near) complete genomes by means of a phylogenetic tree and pairwise distance calculation. As seen with the partial ORF2 sequences, the Swiss 3h sequences formed a distinct cluster, branching into two subclusters. The pairwise distance calculation confirmed the four new Swiss sequences to be assigned to subtype 3h. However, the three wild boar sequences seem to be not only more distantly related to the previously described 3h_s reference genomes but also to the classic 3h sequences from France (Figure 5). It will be interesting to see if the assignment to subtype 3h will hold true for future sequences of the Swiss cluster. Since the three clusters within subtype 3h are not only genetically distinct but differ also regarding geographical distribution and epidemiology it may be helpful to use a specific nomenclature, e.g., including the names of the formerly proposed subtypes, for molecular epidemiological purposes.

## 5. Conclusions

We have confirmed the predominance of a Swiss-specific cluster within HEV subtype 3h in Swiss pigs and meat products and shown that pigs and wild boars share the same subtypes. The cluster 3h_s, which may result from local virus evolution due to the isolated Swiss pig industry, has so far only been reported in Switzerland. Hence, its determination enables differentiation of domestic and imported infections with hepatitis E virus. Therefore, assignment of HEV sequences into epidemiologically related clusters below the subtype level can be important. However, while the diversity within 3h_s was high, more data is necessary to conclude the farm specificity of single sequences and the suitability of the partial ORF2 sequence for molecular tracing of HEV in Switzerland. 

## Figures and Tables

**Table 1:** HEV real-time RT-PCR (italic) and antibody ELISA results of wild boars from different Swiss cantons. Schaffhausen = SH, Ticino = TI, Aargau = AG, Zurich = ZH, Basel-Landschaft = BL, Solothurn = SO.

Canton	Material	Test	No. Tested	No. Positive	% Positive
SH	*Liver*	*real-time RT-PCR*	*75*	*7*	*9.3*
	Liver juice	Ab-ELISA	75	21	28.0
	Diaphragm juice	Ab-ELISA	141	23	16.3
TI	*Liver*	*real-time RT-PCR*	*46*	*0*	*0.0*
	Liver juice	Ab-ELISA	46	3	6.5
AG	Diaphragm juice	Ab-ELISA	87	4	4.6
ZH	Diaphragm juice	Ab-ELISA	64	10	15.6
BL	Diaphragm juice	Ab-ELISA	92	7	7.6
SO	Diaphragm juice	Ab-ELISA	6	0	0.0
Unknown	Diaphragm juice	Ab-ELISA	55	1	1.8
**Total**		***real-time RT-PCR***	***121***	***7***	***5.8***
		**Ab-ELISA**	**566**	**69**	**12.2**

**Table 2:** Overview of the samples with successful partial ORF2 sequencing, the year of sampling, real-time RT-PCR Ct values, the subtype allocation and the NGS results.

Origin	Sample ID	Year	Material	HEV Ct Value	HEV Subtype	NGS Reads ^1^	NGS Coverage ^1^ (%)
Wild boars	WB12	2017	Liver	37.7	3l	7	12.6
	WB33	2018	Liver	24.3	3h_s ^2^	6304	99.3
	WB36	2018	Liver	28.9	3h_s	686	95.6
	WB39	2018	Liver	34.4	3h_s	156	72.8
	WB40	2018	Liver	33.7	3h_s	254117	100
	WB74	2019	Liver	33.5	3h_s	283	70.7
Pig SLH	PL148	2018	Liver	37.3	3h_s	0	0.0
Pigs CCP	HD1	2018	Liver	32.3	3h_s	174	88.1
	HD8	2018	Liver	37.2	3l	2	4.0
	HD12	2018	Liver	35.7	3h_s	114	74.4
	HD22	2018	Liver	32.0	3h_s	155	82.7
	KW13L	2018	Liver	27.6	3h_s	257	89.1
	KW13F	2018	Feces	21.5	3h_s	6775	98.2
	KW20	2018	Liver	33.3	3h_s	0	0.0
	KW22	2018	Liver	29.4	3h_s	155	82.7
Meat products	BLV00672	2016	Coarse liver paté	28 ^3^	3c	0	0.0
	BLV00691	2016	Smooth liver paté	27	3c	14	23.4
	BLV00735	2016	Liver salsiz	38	3h_s	0	0.0
	BLV00754	2016	Mortadella di fegato	37	3h_s	n.d.	n.a.
	BLV00761	2016	Mortadella di fegato	29	3h_s	n.d.	n.a.
	BLV00916	2017	Smooth liver paté	33	3h_s	n.d.	n.a.
	BLV01126	2018	Deer salsiz	34	3h_s	n.d.	
	BLV01189	2019	Saucisse aux choux	32	3h_s	0	0.0
	BLV01185	2019	Saucisse aux choux	32	3h_s	1	1.4
	16LA03650	2016	Mortadella di fegato	34	3h_s	0	0.0
	16LA05705	2016	Mortadella di fegato	31	3h_s	0	0.0

^1^ Highest number of quality controlled NGS reads aligning to a HEV reference genome and the respective genome coverage of this reference. ^2^ Swiss cluster within subtype 3h according to phylogenetic analyses. ^3^ Ct values of meat products as provided by the laboratory of initial testing.

## Data Availability

The sequences generated in this study are available on NCBI GenBank (https://www.ncbi.nlm.nih.gov/genbank/, accessed on 20 October 2021) under the accession numbers MZ923532–MZ923556. NGS raw data used to generate the four full-length sequences are deposited in NCBI Sequence Read Archive (SRA) as BioProject PRJNA772545 (BioSamples SAMN22376915 to SAMN22376918). Supplementary material showing detailed results of this study are available online.

## Supplementary Materials

The following Supplementary Materials are available online at https://www.mdpi.com/article/10.3390/ani11113177/s1, Figure S1: Map of Switzerland, Figure S2: NGS coverage pattern, Figure S3: Map of the hunting grounds in canton Schaffhausen; Table S1: List of all food samples, Table S2: Results and information of all samples from carcass collection points, Table S3: Results and information of wild boar livers from canton Schaffhausen, Table S4: Matrix list of the pairwise distance values of all 536 sequences.
