# Supplementary material for: Genetic Diversity of Hepatitis E Virus Type 3 in Switzerland—From Stable to Table
Source: Animals (Basel). 2021 Nov 7;11(11):3177. doi: 10.3390/ani11113177 (PMC8614342; doi:10.3390/ani11113177)

**Figure S1**. Map of Switzerland indicating the location of slaughterhouses (orange), carcass collection points (turquoise) and cantons where wild boar samples originated from (blue). AG = Aargau, BL = Basel-Landschaft, SH = Schaffhausen, SO = Solothurn, TI = Ticino, ZH = Zürich. Map created with MS excel.

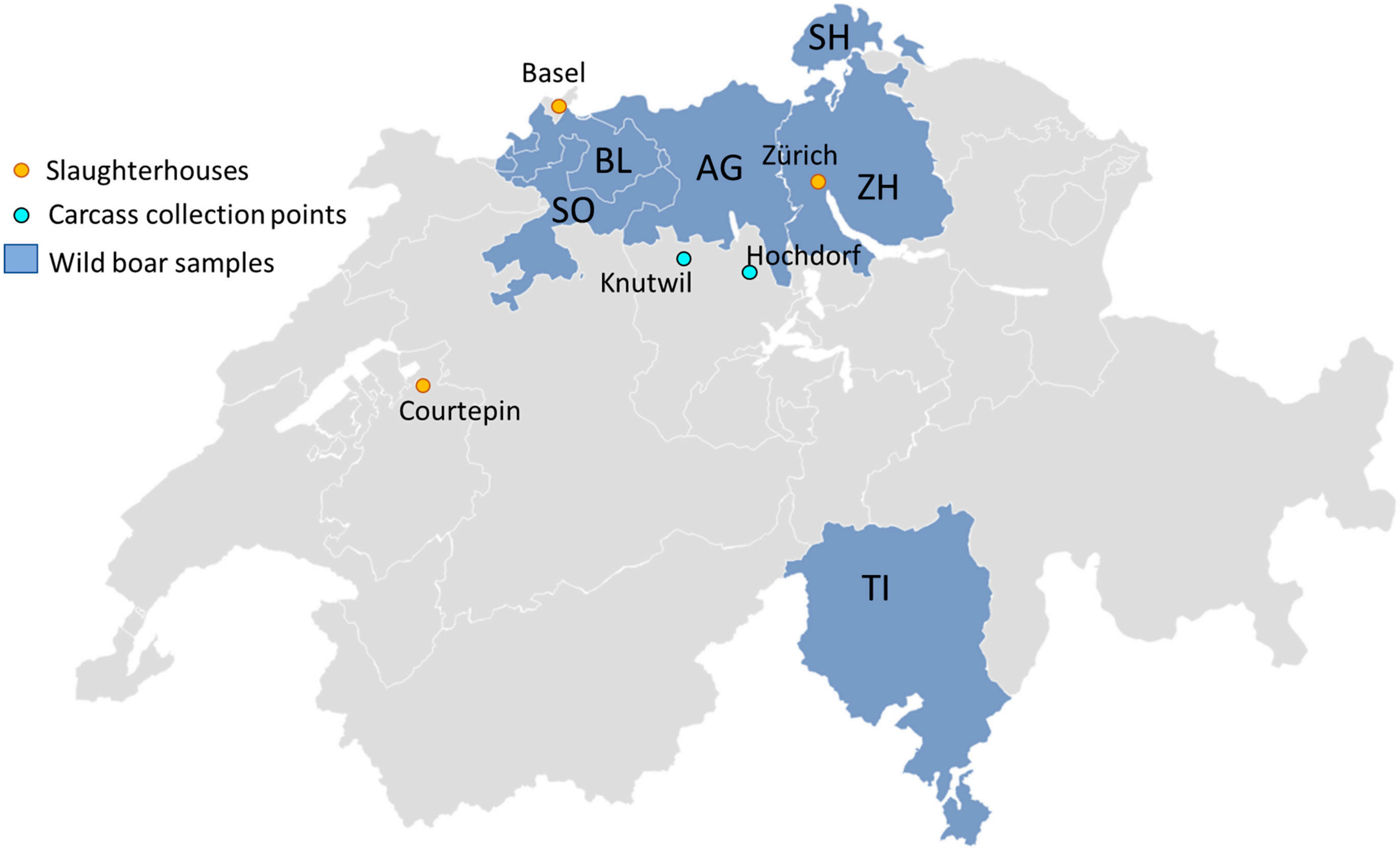

Supplement: Supplementary file 1 [file animals-11-03177-s001.zip › Supplementary Figure S1.pdf]
