# Supplementary material for: Genetic Diversity of Hepatitis E Virus Type 3 in Switzerland—From Stable to Table
Source: Animals (Basel). 2021 Nov 7;11(11):3177. doi: 10.3390/ani11113177 (PMC8614342; doi:10.3390/ani11113177)

Figure S3. Map of the hunting grounds in canton Schaffhausen. The borders of hunting grounds where we received livers from are in black, all other borders of hunting grounds are in orange. The name of hunting grounds with HEV RNA positive wild boars are written in red for HEV-3h_s cluster viruses and in blue for HEV-3l. Map and borders are derived from the public geographic information site of the canton Schaffhausen: https://map.geo.sh.ch/geoportal/.

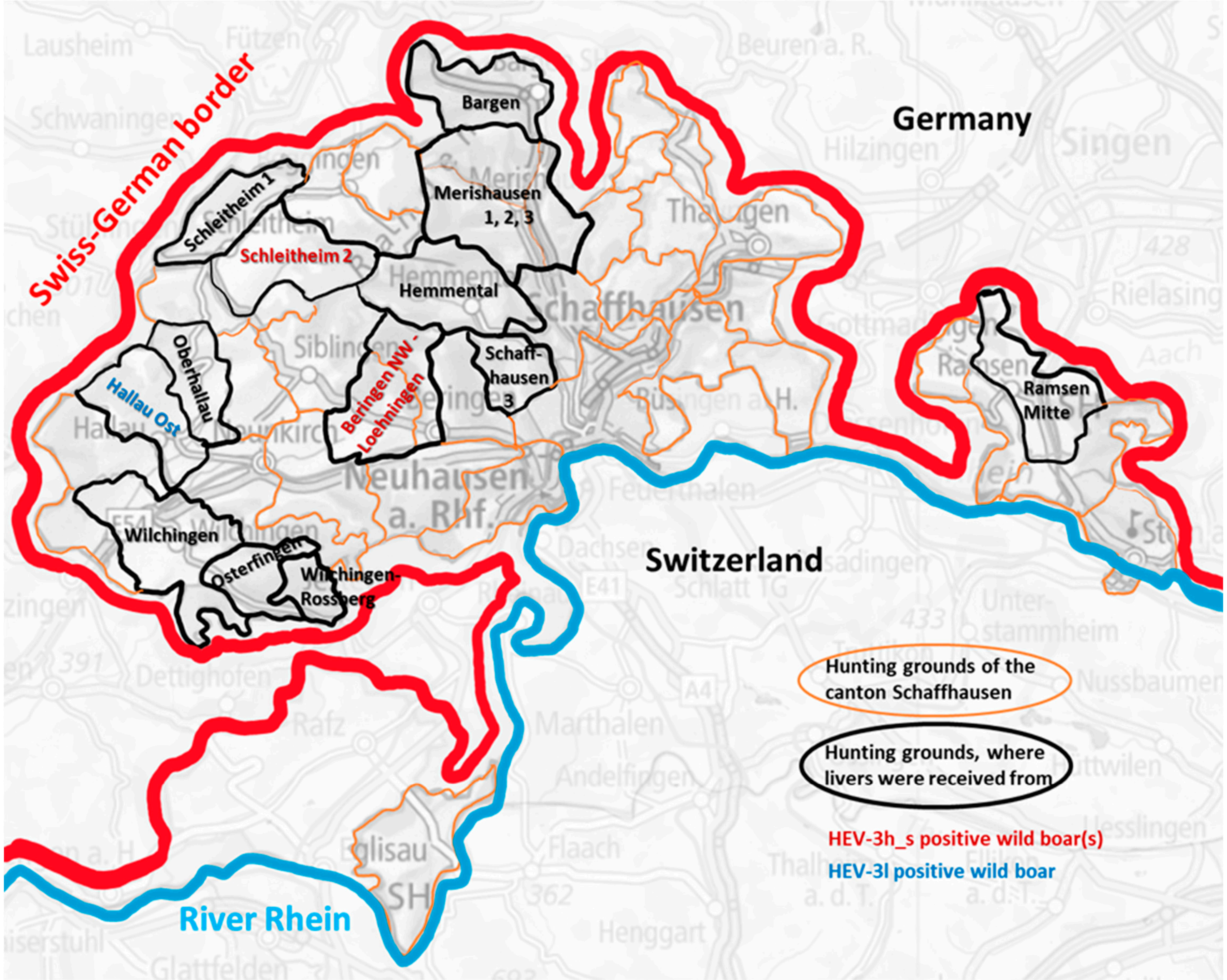

Supplement: Supplementary file 1 [file animals-11-03177-s001.zip › Supplementary Figure S3.pdf]
