# Supplementary figures and images for: Genetic Diversity of Hepatitis E Virus Type 3 in Switzerland—From Stable to Table
Source: Animals (Basel). 2021 Nov 7;11(11):3177. doi: 10.3390/ani11113177 (PMC8614342; doi:10.3390/ani11113177)

**Figure S2.** Coverage pattern of NGS reads to reference genomes (highlighted with squares).

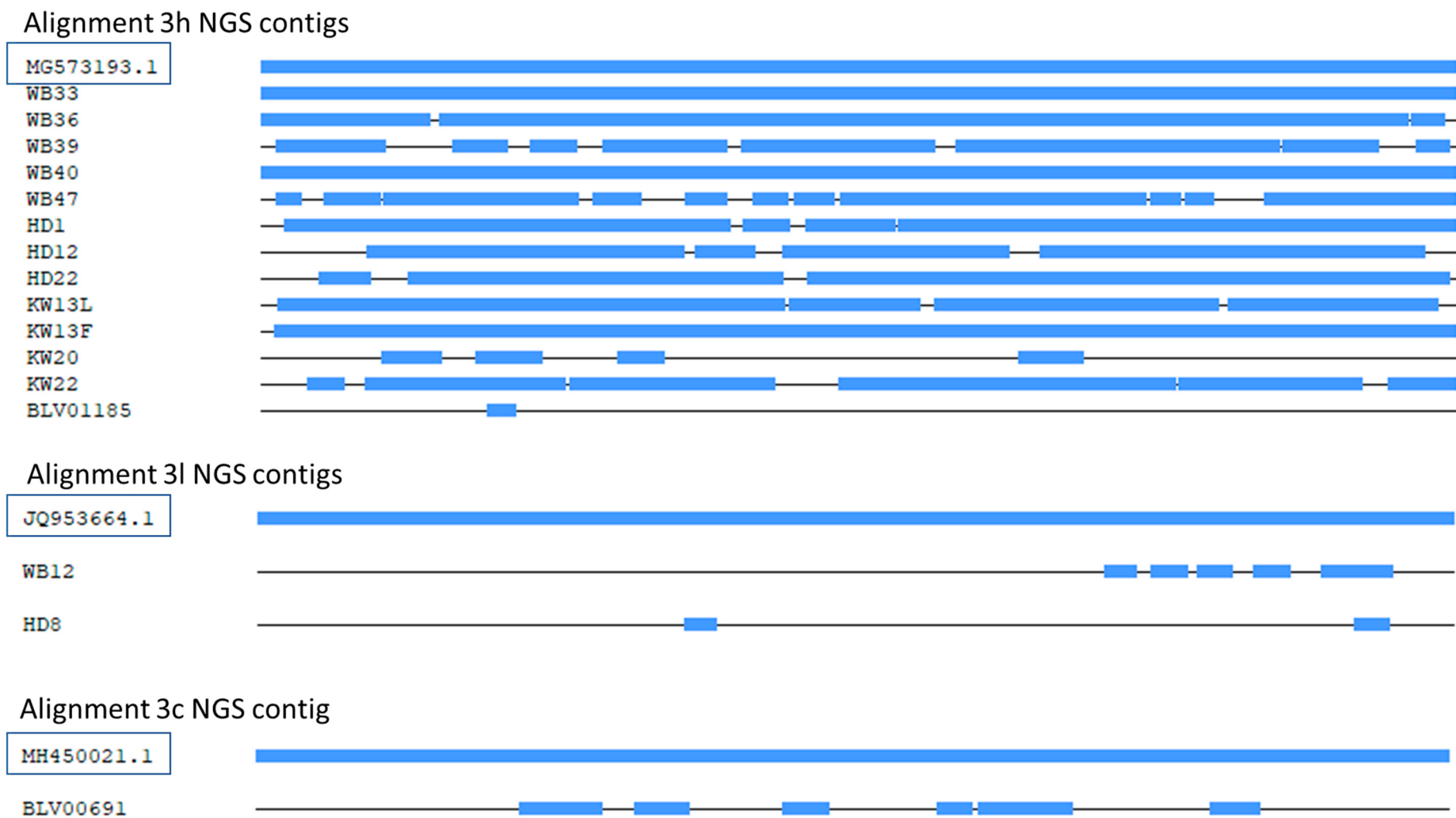

Supplement: Supplementary file 1 [file animals-11-03177-s001.zip › Supplementary Figure S2.pdf]
